# Implementation of BMP Strategies for Adaptation to Climate Change and Land Use Change in a Pasture-Dominated Watershed

**DOI:** 10.3390/ijerph9103654

**Published:** 2012-10-15

**Authors:** Li-Chi Chiang, Indrajeet Chaubey, Nien-Ming Hong, Yu-Pin Lin, Tao Huang

**Affiliations:** 1 Department of Bioenvironmental Systems Engineering, National Taiwan University, 1, Section 4, Roosevelt Road, Da-an District, Taipei City 106, Taiwan; Email: lchiang@ntu.edu.tw (L.-C.C.); jay7753@hotmail.com (T.H.); 2 Department of Agricultural and Biological Engineering, Purdue University, 225 South University Street West Lafayette, IN 47907, USA; Email: ichaubey@purdue.edu; 3 Environment and Energy Management Center, Overseas Chinese University, No. 100, Chiao Kwang Road, Taichung 407, Taiwan; Email: hong@ocu.edu.tw

**Keywords:** best management practice (BMP), climate change, land use change, soil and water assessment tool (SWAT), nonpoint source pollution

## Abstract

Implementing a suite of best management practices (BMPs) can reduce non-point source (NPS) pollutants from various land use activities. Watershed models are generally used to evaluate the effectiveness of BMP performance in improving water quality as the basis for watershed management recommendations. This study evaluates 171 management practice combinations that incorporate nutrient management, vegetated filter strips (VFS) and grazing management for their performances in improving water quality in a pasture-dominated watershed with dynamic land use changes during 1992–2007 by using the Soil and Water Assessment Tool (SWAT). These selected BMPs were further examined with future climate conditions (2010–2069) downscaled from three general circulation models (GCMs) for understanding how climate change may impact BMP performance. Simulation results indicate that total nitrogen (TN) and total phosphorus (TP) losses increase with increasing litter application rates. Alum-treated litter applications resulted in greater TN losses, and fewer TP losses than the losses from untreated poultry litter applications. For the same litter application rates, sediment and TP losses are greater for summer applications than fall and spring applications, while TN losses are greater for fall applications. Overgrazing management resulted in the greatest sediment and phosphorus losses, and VFS is the most influential management practice in reducing pollutant losses. Simulations also indicate that climate change impacts TSS losses the most, resulting in a larger magnitude of TSS losses. However, the performance of selected BMPs in reducing TN and TP losses was more stable in future climate change conditions than in the BMP performance in the historical climate condition. We recommend that selection of BMPs to reduce TSS losses should be a priority concern when multiple uses of BMPs that benefit nutrient reductions are considered in a watershed. Therefore, the BMP combination of spring litter application, optimum grazing management and filter strip with a VFS ratio of 42 could be a promising alternative for use in mitigating future climate change.

## 1. Introduction

Best management practices (BMPs) are often used to control the losses of non-point source (NPS) pollutants to receiving water bodies. Selection of BMPs is specific to topographic, soil, land use, and climate conditions. For example, NRCS recommended using phosphorus index (PI) rating, soil phosphorus threshold values, or soil test to establish acceptable phosphorus application rates [1]. Different approaches to mitigate animal waste problems were adopted in the Rural Clean Water Program (RCWP) projects conducted in the American states of Utah and Florida due to their different climatic characteristics [2]. Multiple BMPs are usually combined together in a watershed, such as tillage and nutrient management practices [3] or grazing management and vegetative buffers [4] to effectively control pollutants from various sources. When numerous BMP options are available, whether various combinations of BMPs work synergistically or cancel the effect of each other when implemented together in a watershed must be evaluated [5,6,7]. 

The Conservation Effects Assessment Project (CEAP) was initiated by the USDA Natural Resources Conservation Service (NRCS), Agricultural Research Service (ARS), and Cooperative State Research, Education and Extension Service (CSREES) (now National Institute of Food and Agriculture or NIFA) to evaluate the effects of conservation practices at the watershed scale and to estimate the impacts of conservation practices at national and regional levels. One of the efforts of CEAP is to measure long-term watershed-specific effects of conservation practices on environmental quality. This task is performed by examining changes in water quality for a specific period when a watershed has undergone certain conservation management practices. For example, 70% and 41% of the reductions in suspended sediment concentration and total phosphorus, respectively, were found as a result of conservation practices in the Beasley Lake watershed, Mississippi, from 1995 to 2005 [8]. Increased levels of residue cover were found to be negatively correlated with nutrient concentrations and loads in the St. Joseph River watershed in Indiana, where multiple tillage management was implemented from 2006–2007 [9].

In addition to field investigation of the impacts of conservation practices, watershed models are a highly efficient means of evaluating how various combinations of BMPs can improve water quality and reduce NPS losses. These models are used to make watershed response predictions in two modes based on the time scale of interest: futurecast and hindcast. In futurecast, a watershed model is used to evaluate how various BMPs likely improve water quality. For example, Yuan [10] identified the critical areas where conservation practices must be implemented in the Mississippi Delta Beasley watershed by using the annualized agricultural non-point source (AnnAGNPS) model. According to their results, converting all crop lands to no-till soybeans or cotton reduces sediment losses by 64–77% over current conditions. By using the soil and water assessment tool (SWAT), Chaubey [5] evaluated the effectiveness of 171 BMP scenarios in a CEAP watershed from 2004–2028, as represented by 250 projected weather variations. Hindcast studies adopt a watershed model to retrospectively evaluate how much water quality was impacted by current conservation practices or how much water quality would have improved if certain suites of BMPs had been previously implemented in a watershed. For example, from 2003–2006, conservation practices reduced sediment, total nitrogen and phosphorus losses by 69%, 46% and 49%, respectively in the Upper Mississippi River Basin [1]. Locke [8] suggested that no-tillage practices could reduce sediment loadings to a range of 15%–69% of the existing condition in the Mississippi Delta region, based on AnnAGNPS model simulations. By using the soil and water assessment tool (SWAT), Bracmort [11] quantified the long-term impacts of various structural BMPs on sediment and phosphorus losses over a 25-year period (1975–2000). That study also estimated that current BMPs reduced sediment and phosphorus yield by 7–10% and 7–17%, respectively.

Designing a futurecast model often involves making climate change scenarios or land use change scenarios, which could be the main source of uncertainty in the evaluation of conservation practices [12,13,14]. However, hindcast modeling, which utilizes climate data and historical land use data, likely has less uncertainty and can be used to evaluate the effectiveness of current conservation practices and how water quality is improved even if another suite of BMPs has been implemented in the watershed. Additionally, the hindcasted results could be used as a baseline to evaluate how simulated future conditions impact watershed responses [15]. Therefore, this study evaluates the effectiveness of various combinations of management practices in improving water quality for the periods of 1992–2007 and 2010–2069 in the Lincoln Lake watershed, Arkansas. Additionally, the amount of pollutants that would have been reduced if the most effective management practices were implemented in the watershed is quantified. Moreover, exactly how climate change impacts water quality improvement at different spatial scales is quantified. By using the SWAT model, 171 pasture management practice scenarios are evaluated, including 19 nutrient management options, three grazing management types, and three vegetated filter strips (VFS). This study was undertaken from 1992–2007 with the implementation of several BMPs in the watershed since 1992. We hypothesized that future climate change impacts the BMP performance in different ways to improve water quality.

## 2. Materials and Methods

### 2.1. Study Area

**Figure 1:**
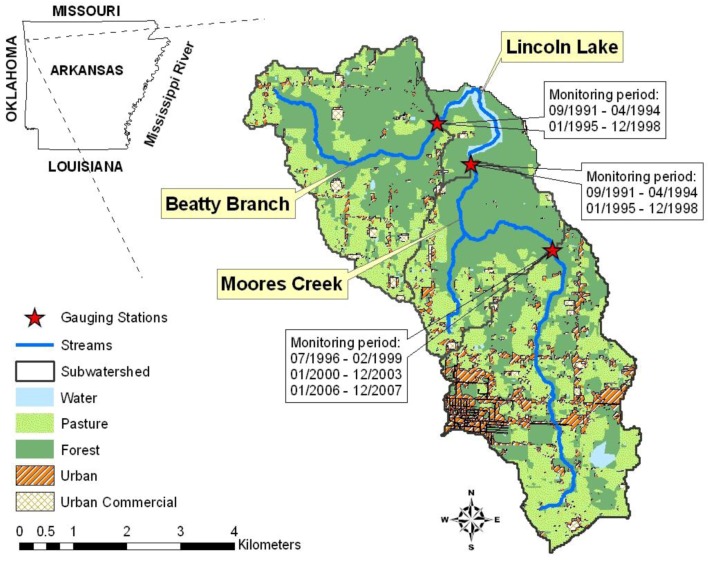
Location of Beatty Branch, Moores Creek, gauging stations with monitoring periods and 2004 land use distribution in the Lincoln Lake watershed.

**Figure 2:**
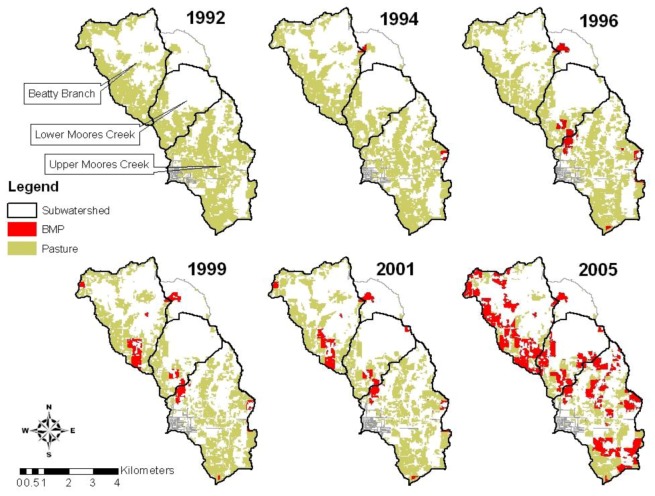
Location of BMPs on pasture lands in the Lincoln Lake watershed from 1992 to 2005.

This study was conducted in the Lincoln Lake watershed, a 32 km^2^ agricultural watershed within the Illinois River basin located in Northwest Arkansas and Eastern Oklahoma. Moores Creek and Beatty Branch are the two major tributaries in the watershed, representing 21 and 11 km^2^ of the watershed area, respectively. This watershed is one of the 13 watersheds in the Conservation Effectiveness Assessment Project (CEAP) funded by the USDA-CSREES. The Lincoln Lake watershed was a pasture dominated watershed where pasture lands constituted more than 48% of the entire watershed in 1992. However, due to rapid urbanization in the past 15 years, pasture lands have decreased with a concurrent increase in urban lands. Table 1 shows the land use distribution in the Lincoln Lake watershed from 1992 to 2004. In 2004, pasture, forest, urban residential and urban commercial represented 36%, 49%, 12% and 2% of the watershed area, respectively (Figure 1). There are numerous poultry, beef, and dairy cattle production facilities in the pasture fields of the watershed. High levels of fertilizer and manure usages on perennial forage crop production in the watershed have been shown to increase surface and ground water pollution due to inputs of sediment, nutrients and pathogens [16]. Since 1994, areas that had BMPs implemented in the watershed have increased from 1% to 34% of the entire watershed area, representing 53% of total pasture areas in the watershed in 2005 (Figure 2). BMPs were first implemented in the northeast part of the Beatty Branch and Upper Moores Creek subwatersheds in 1994. The western portion of the Beatty Branch and northeastern portion of the Lower Moores Creek subwatershed had BMPs implemented later in 1999. In 2005, most of the pasture lands had at least one BMP implemented, except the southwestern part of the Moores Creek subwatershed. Recommendations for BMP implementation have changed over the years from poultry litter application based on meeting plant nitrogen demand in the early 1990s to phosphorous based application in 2000. Currently, farmers are encouraged to apply alum-treated poultry litter based on the Arkansas Phosphorus Index to reduce soluble phosphorus concentration in poultry litter [17,18]. 

**Table 1:** Historical land use distribution in the Lincoln Lake watershed from 1992 to 2004. (Note: number in parenthesis denotes the percentage of the watershed).

Land Use	1992	1994	1996	1999	2001	2004
Forest	1,493 (46.4)	1,619 (50.3)	1,574 (48.9)	1,628 (50.6)	1,619 (50.3)	1,567 (48.7)
Pasture	1,532 (47.6)	1,377 (42.8)	1,322 (41.1)	1,229 (38.2)	1,164 (36.2)	1,151 (35.8)
Urban	107 (3.3)	138 (4.3)	225 (7.0)	267 (8.3)	339 (10.6)	381 (11.8)
Urban commercial	27 (0.9)	30 (0.9)	36 (1.1)	39 (1.2)	41 (1.3)	50 (1.5)
Other	58 (1.8)	53 (1.7)	61 (1.9)	55 (1.7)	54 (1.7)	70 (2.2)
Total	3,217	3,217	3,217	3,217	3,217	3,217

Three monitoring sites were located at the Beatty Branch, Lower Moores Creek and Upper Moores Creek with different monitoring periods depending on the monitoring projects funded in the watershed (Figure 1). Nutrient and sediment transport were first monitored from September 1991 to April 1994 at Beatty Branch (BB) and Lower Moores Creek (LMC). Vendrell [19] concluded that the BMPs were able to retard nitrogen transport as indicated with a decrease in mean concentrations of ammonia nitrogen (NH_4_-N) and Total Kjeldahl Nitrogen (TKN) from January 1995 through December 1998 at the Lower Moores Creek and Beatty Branch sites, and from July 1996 through February 1999 at the Upper Moores Creek site, respectively. Nelson [20] compared data from 2006 to 2007 with the data from 2000 to 2003, and the results showed that TP concentration dropped nearly 50% (0.19 mg/L in 2000 and 0.1 mg/L in 2007) and nitrate-nitrogen declined by 66% (3.57 mg/L in 2000 and 1.21 mg/L in 2007) indicating that implemented BMPs resulted in reduced nutrient loads in the watershed.

### 2.2. Model Description and Input Data Preparation

The Soil and Water Assessment Tool (SWAT) model was used to estimate and hindcast the effectiveness of various management practice combinations on water quality considering the dynamic land use and management conditions in the watershed. The model can predict long-term impacts of land use and management on water, sediment and agricultural chemical yields at different scales in a mixed land use watershed [21]. The SWAT model has been widely used to quantify the linkage between BMPs and water quality at the watershed-scale in the United States [22]. The major GIS input files for the SWAT model were the digital elevation model (DEM) at 30 m resolution [23], land use and land cover [24] and SSURGO soil data. The land use maps for the years 1992, 1994, 1996, 1999, 2001 and 2004 were developed using moderate spatial resolution (28.5 m × 28.5 m) Landsat Thematic Mapper (TM) satellite images [25]. The watershed was delineated into 72 subbasins based on the DEM, specification of streams and inlets/outlets. Then the sub-basins were portioned into homogeneous units (hydrologic response units, HRUs) by setting 0% threshold percentages of land use and soil type for accurately capturing the land use change that occurred between 1992 and 2004. Weather data (daily precipitation, minimum and maximum temperature) were obtained from Fayetteville Weather Station located approximately 25 km from the watershed. Other weather variables needed by the model (solar radiation, wind speed and relative humidity) were estimated using the weather generator built into the SWAT model.

The pasture management information was collected by Washington County extension personnel and used to calculate the area-weighted average of fertilizer and manure rates for representing the existing land application and grazing management at each HRU [26]. The calculated fertilizer and manure rates, as well as grazing periods were built into the management files for pasture HRUs as SWAT input files. There were seven types of manure and fertilizers applied in the Lincoln Lake watershed. The inorganic fertilizers included urea, anhydrous ammonia, and triple 17 (17% of N, P_2_O_5_ and K_2_O). Organic manure included beef-fresh manure, hen/pullet manure, broiler-fresh manure, and alum-treated broiler manure. The SWAT model has the ability to define specific types of manure and fertilizers by providing detailed fertilizer and manure components, such as fractions of mineral N (or P), organic N (or P), and a ratio of ammonium to mineral N in the SWAT fertilizer database. It should be noted that the nutrient content of broiler-fresh manure and alum-treated broiler manure were based on poultry houses located in the northwest Arkansas [27,28]. The pasture management information including amount of litter and fertilizer application, timing of manure and fertilizer application, and grazing intensity and dates were obtained from a detailed review of historical nutrient management plans and interviews with 63 out of 75 farmers located in the watershed [26]. The timing and amount of litter and fertilizer application varied in this watershed during 1992–2004. The average litter application and approximate dates of application were 2,500 kg/ha applied each time on 30 April and 31 August. The daily dry weight of forage intake by grazing animals was 10.14 kg/ha/day and the dry mass of the manure excreted ranged from 0.01–14.2 kg/ha/day. The number of days animals grazed in the watershed ranged from 11–365 days/year. Detailed information of management practices and schedules for SWAT management files can be found in Chaubey [5].

In order to incorporate the dynamic land use change, the SWAT 2009 model which incorporates HRU fraction changes was used. More details about developing HRU fraction files for individual years and how the SWAT model reads the different HRU fraction files can be found in Chiang [27]. One of the major changes in the SWAT 2009 model is to simulate vegetated filter strips (VFS) by using the VFS ratio of pollutant sourcing area to the VFS area and including the impact of concentrated flow. In the 2005 executable version of SWAT, the model simulates the performance of VFS using the VFS width alone. Several factors may influence the removal rate of chemicals through the buffers, such as forms of chemicals (soil-bound or soluble), length/width of buffer strips, vegetation types, hydrologic conditions and soil types [29,30,31]. Many studies have focused on simulating the sediment trapping efficiency of filter strips at the field scale, where runoff was distributed fairly uniformly over the buffer area [32,33]. However, numerous studies indicate that non-uniform flow is more common than uniform flow passing through the buffer area [34,35,36,37]. When a buffer is designed, it should be based on a ratio of upslope contributing area to effective buffer area rather than on buffer dimensions (width, length, shape, total area) alone [38,39]. Therefore, the VFS ratio is critical, especially when high loads or concentrated flow conditions exist in the VFS areas [31]. In SWAT 2009, VFS is separated into two sections during the simulation. Section one is 90% of the VFS that receives the least flow, and section two is the other 10% of the VFS that receives 25–75% of the flow [40]. Runoff and sediment loadings before passing through the VFS are calculated using the ratio of the drainage area to VFS area (DAFS_ratio_) for each VFS section and HRU runoff and sediment yield prediction. Sequentially, the reduction of runoff, sediment and nutrients are calculated as follows [40]:

R_R_ = 75.8 − 10.8 ln(R_L_) + 25.9 ln(K_SAT_) (1)

where R_R_ is the runoff reduction (%); R_L_ is the runoff loading (mm); and K_SAT_ is the saturated hydraulic conductivity (mm/h).

S_R_ = 79 − 1.04 S_L_ + 0.213 R_R_ (2)

where S_R_ is the sediment reduction (%); S_L_ is the sediment loading (kg/m^2^); and R_R_ is the runoff reduction (%).

TN_R_ = 0.036 S_R_^1.69^ (3)

where TN_R_ is the total nitrogen reduction (%); and S_R_ is the sediment reduction (%).

TP_R_ = 0.9 S_R_ (4)

where TP_R_ is the total phosphorus reduction (%); and S_R_ is the sediment reduction (%).

The SWAT outputs include flow and water quality information at different temporal (daily, monthly and annual) and spatial (HRU, subbasin, watershed) scales. In this study, monthly flow, sediment, nitrogen and phosphorus loads at the gauging station were of interest for model calibration and validation. The annual pollutant loads at a subbasin were calculated by multiplying the annual area-averaged pollutant loads from a HRU by the HRU area, and then summing the annual loads at each HRU in the subbasin. These processed outputs were used to quantify the amount of pollutant losses if a certain suite of BMPs was implemented in the watershed. 

### 2.3. SWAT Model Calibration and Validation

Sensitivity analysis is usually performed to identify which parameters in a model most influence outputs of interest. Based on the sensitivity analysis results and the identified calibration parameters in several SWAT publications, 13 parameters were modified for calibrating flow, sediment, nitrogen and phosphorus in this study [11,41]. Model calibration and validation were performed for monthly stream flow, TSS, TN and TP using the measured flow and water quality data collected at the Upper Moores Creek for the period 01/1996–2/1999, 01/2000–12/2003 and 01/2006–12/2007. Measured stream flow data were available for 8 years, while water quality data (TSS, TN and TP) were only available for seven years, which was the same monitoring period as flow except the year of 1996. In order to make the model comprehensively capture the watershed responses, we selected 01/2001–12/2003 and 01/2006–12/2007 as the model calibration period due to major land use changes that occurred during this period. Subsequently, we selected 01/1996–2/1999 and 1/2000–12/2000 as the model validation period for flow and 01/1997–2/1999 and 1/2000–12/2000 for TSS, TN and TP. 

Flow was calibrated first because it can influence other outputs [41] and it has less measurement uncertainty [42]. Flow calibration was followed by sediment calibration because organic nitrogen and phosphorus are usually attached on sediment and transported in runoff [43,44]. Two quantitative statistics used for model evaluation were Nash-Sutcliffe efficiency (NSE) [45] and coefficient of determination (R^2^). The Nash-Sutcliffe efficiency (NSE) is a normalized statistic indicating how well the observed and predicted data fit the 1:1 line [45]. Predicted results with an NSE value greater than 0.5 is regarded as satisfactory [46]. The coefficient of determination (R^2^) describes the portion of the variance in the measured data that are explained by the model. The greater R^2^ values indicate a less error variance and a model with R^2^ value greater than 0.5 is usually considered acceptable [47,48]. 

The process of calibration was repeated by adjusting the parameters and computing NSE and R^2^ between observed and predicted data to ensure that in optimizing one variable, other variables were not substantially influenced [41]. To test if the parameters were appropriately selected for model calibration, model validation was performed for evaluating the accuracy of the model by comparing simulation results to a different set of observation data from the calibration dataset. 

### 2.4. Management Practice Scenarios

The watershed management practice scenarios considered in this study were grouped into three categories: grazing management, vegetated filter strips (VFS), and nutrient management. These scenarios were based on detailed interactions with the watershed stakeholders and history of past BMPs implemented in the watershed [26]. 

**Grazing management:** Three grazing intensities were considered: (1) no grazing; (2) optimum grazing; and (3) overgrazing. Based on detailed discussion with the county extension experts, the minimum plant biomass for grazing to occur was set at 2,700 and 1,009 kg/ha, respectively, for optimum and overgrazing (Ron Morrow, personal communication). The overgrazing application started on 30 September and lasted for 213 days until 30 April of the next year. Typically, optimum grazing comprises rotating grazing animals through various HRUs such that a minimum biomass is maintained in the field. Based on information on typical optimum grazing management, it was assumed that within 30 days the cattle should graze through the whole watershed and would stay for approximately 4–6 days in each pasture HRU [49]. This approach was similar to grazing operations reported in other watersheds located near the study area [41]. 

**Vegetated filter strips (VFS):** Vegetated filter strips (VFS) have been proven to be an effective management practice for trapping sediment and nutrients in field runoff [50,51,52]. The Natural Resources Conservation Service (NRCS) has developed a method to design and estimate sediment removal from VFS using the Revised Universal Soil Loss Equation, Version 2 (RUSLE2) [53] in which VFS are designed to have a minimum 10-year life time and the VFS need to be re-established once the sediment accumulation reaches 6 inches [54]. NRCS developed an equation to calculate the number of years to reach 6 inches at the annual sediment accumulation rate. The annual sediment accumulation rate is calculated as follows:

Rate_sed_ = Sed × 21.74(ft^3^/ton) × Trap_sed_ × VFS_ratio_ ×43,560(ft^2^/acre) × 12(in/ft) (5)

where Rate_sed_ is the annual sediment accumulation rate (in/year); Sed is sediment delivery to VFS (tons/acre/year); Trap_sed_ is the trapping efficiency; and VFS_ratio_ is the ratio of contributing field area to VFS area.

In this study, the life time of the VFS was assumed to be 25 years, and different levels of trapping efficiency were set equal to 29%, 56% and 100% based on various studies in the Southeastern U.S. region [7,37,55]. The maximum annual sediment loads (2,333 kg/ha/year), which were estimated with 250 weather realizations during a 25-year simulation period in previous study [5], were selected to design VFS ratios with three levels of trapping efficiency under the worst condition of sediment delivery. The VFS ratios were calculated inversely using Equation (5). Thus, VFS ratios were designed to be 146, 76 and 42, respectively, with the trapping efficiency of 29%, 56% and 100% to maintain a VFS functional period of 25 years. In other words, when the VFS ratio is 42 at any trapping efficiency, it will take at least 25 years to accumulate 150 mm in the VFS. Similarly, when the VFS ratio is 76, the VFS can function for 14–48 years with the trapping efficiency 100%–29%. However, if the VFS ratio is 146 with greater than 82% trapping efficiency, the life time of VFS will be shorter than 9 years. Therefore, we selected VFS ratios of 42 and 76 as two different levels of VFS that could be implemented in the watershed. 

**Nutrient management:** Nutrient management scenarios evaluated in this study included various poultry litter application rates, litter characteristics, and application timing. DeLaune [18] suggested that the litter application in pasture areas should not exceed 4.94 t/ha for warm season grasses, and 7.41 t/ha for cool season grasses in nutrient surplus watersheds in Northwest Arkansas. Therefore, the litter application rates evaluated were 2.47, 3.71 and 4.94 t/ha in spring (applied on 30 Apri) and summer (31 August) to support growth of warm season grasses, and 4.94, 6.18 and 7.41 t/ha in fall (15 October) to support growth of cool season grasses. For all application rates and timings evaluated in this study, two types of poultry litter were selected-normal poultry litter and alum-amended litter. Many studies have shown that the alum-amended litter was able to reduce P losses in surface runoff and leaching [51,56,57]. Additionally, alum in poultry litter was shown to increase yields due to greater N mineralization and less NH_3_ emissions [57,58]. The total number of nutrient management scenarios evaluated was 18 (3 nutrient application rates × 3 application timings × 2 litter types). Management practice scenarios which consist of no litter application with any grazing management and VFS implementation were also added for further comparison. 

The combination of above management practices resulted in 171 different management practice scenarios. The pasture management practices that existed in the watershed during 1992–2007 were regarded as baseline (scenario 172) and were used to compare the effectiveness of selected management practice combinations in reducing NPS pollutants of concern from the watershed.

### 2.5. Climate Change Scenarios

The future 100-year climate condition with no climate change was first generated using the historical climate data from 1970 to 1999. This no climate change (NCC) condition retains the same statistical characteristics as the historical climate data. Three GCMs (general circulation models) simulations were used to generate short-term (2010–2039) and mid-term (2040–2069) climate change conditions. Those GCMs are CCSM (National Center for Atmospheric Research, NCAR, Community Climate System Model, version 3.0), CGCM2 (Meteorological Research Institute, Japan Meteorological Agency, MRI-CGCM2.3.2), and GFDL21 (Geophysical Fluid Dynamics Laboratory, NOAA, CM2.1). All the data for the GCMs were obtained from the Data Distribution Centre of the Intergovernmental Panel on Climate Change (IPCC). Since the spatial resolutions of GCMs are too coarse to represent local climate characteristics in the watershed, the technique of simple downscaling between the baseline and the climate scenario of the nearest GCM grid was applied directly. 

The future change in temperature in the study area is assumed to be the same as the difference between temperatures simulated using GCMs for the future and current conditions at the weather station [59,60]: 


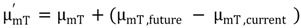
(6)

where and are the current and future mean monthly temperature (°C) respectively; and and are, respectively, the simulated mean monthly temperature (°C) under the current and future scenarios (the annual average for 2010–2039) climate conditions respectively. 

The future change in precipitation in this study area is assumed to be the ratio of the precipitation for the future condition to that for the current condition [60]:


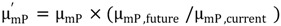
(7)

Where μ_mP_ and μ*'*_mP_ are the current and future mean monthly precipitation (cm), respectively; and μ_mP,current_ and μ_mP,future_ are the simulated mean monthly precipitation (cm) under the current and future climate conditions, respectively. 

We utilized Tung and Haith’s [61] weather generation model to generate daily temperature and precipitation data for the target climate scenarios. In order to produce as many combinations of weather variability as possible, a total of 100 years of daily weather data were generated for the baseline and climate scenarios. Table 2 lists the annual average temperature and precipitation for each climate change scenario. 

### 2.6. Selection of the Best and Worst Pasture Management Practice Combinations

The management practices with the maximum constituent losses is regarded as the worst management practice combination for water quality improvement, while the management practices having the minimum losses is regarded as the best management practice combination. The best and worst scenarios are not the same for all pollutants. For example, litter application can increase the biomass production, infiltration and reduce sediment losses, but meanwhile an increase in nutrient inputs on pasture may exceed nutrient demand of the forage and can lead to greater nutrient losses from the watershed.

**Table 2:** The annual average temperature and precipitation. (Note: Historical climate denotes the observed weather data from 1990–2007; NCC denotes no climate change condition).

Scenarios	Max. Temperature	Min. Temperature	Precipitation (mm)
Historical climate	20.7	9.3	1,245.5
NCC	20.2	8.5	1,105.4
CCSM_S	22.4	10.5	1,063.5
CCSM_M	23.6	11.6	1,170.3
CGCM_S	21.4	9.6	1,069.5
CGCM_M	22.2	10.3	1,114.4
GFDL_S	21.4	9.6	1,080.2
GFDL_M	22.8	10.9	995.0

## 3. Results and Discussion

### 3.1. SWAT Model Performance

Table 3 lists the ranges, default values and calibrated values of the SWAT parameters. The ranges of the SWAT parameters were obtained from the literature [11,62]. The model was initially run using the default SWAT parameters, and the monthly predictions from each step of the model calibration were compared with the monthly measured data at the Upper Moores Creek. The default CN values for each land use were decreased by 10%, indicating that the Lincoln Lake watershed has better soil drainage than the general conditions in the SWAT database. 

The ESCO value was decreased from 0.95 to 0.26 to allow for greater evaporation from lower soil layers. Because of a high base flow, the GWQMN value was increased to 3,000 mm to increase deep percolation losses, a condition typical to karst topography in the watershed. NPERCO and PPERCO values were increased because of a low mineral nitrogen loading and low soluble phosphorus loading, respectively. The PHOSKD value was not linearly related to TP, and PHOSKD = 100 was the optimal value for TP calibration. 

**Table 3:** List of calibration SWAT parameters for each output of interest for the Lincoln Lake watershed SWAT model.

Output	SWAT parameters (Unit)	Short Description	Range	Default	Calibrated
Flow	CN	Initial SCS runoff curve number	39–98	75.25	67.725 (−10%)
	ESCO	Soil evaporation compensation factor	0–1	0.95	0.26
	GWQMN (mm)	Threshold depth of water in shallow aquifer	0–5,000	0	3,000
TSS	SLOPE (m/m)	Average slope steepness	0–0.6	0.072	0.036
	USLE_K	USLE equation soil erodibility K factor	0.01–0.65	0.345	0.1725
	ADJ_PKR	Peak rate adjustment factor for sediment routing in the main channel	0.1–2	1	2
TN	NPERCO	Nitrate percolation coefficient	0.001–1	0.2	1
	CMN	Rate factor for mineralization ofactive organic nutrients	0.001–0.004	0.003	0.004
TP	PPERCO	Phosphorus percolation coefficient	10–17.5	10	17.5
	PHOSKD	Phosphorus soil partitioning coefficient	40–300	175	100

The model calibration and validation results were evaluated using Nash-Sutcliffe efficiency (NSE) and coefficient of determination (R^2^) as the model performance criteria (Table 4). For calibration, the NSE and R^2^ values for flow, TSS, TN and TP were equal to or greater than 0.5, which is generally viewed as a satisfactory model performance [46,47,48]. For validation, the performance of the model in simulating the flow and TP was satisfactory, as indicated by NSE and R^2^ values greater than 0.5. Except for one indication of unsatisfactory model performances (NSE = 0.25 and 0.33 for TSS and TN, respectively), the model simulation of TSS and TN was satisfactory. Our results indicated some extremely high measured TSS and TN values that the model could not simulate resulting in the NSE values for TSS and TN lower than 0.5. The NSE values increased to 0.64 and 0.53 for TSS and TN after the outliers (4 measured monthly TSS values ranging from 238–550 kg /ha and 4 measured monthly TN values ranging from 0.73–0.85 kg/ha) were removed. Concurrently, the R^2^ values for TSS and TN increased to 0.75 and 0.73, respectively. Overall, the model calibration and validation were satisfactory based on NSE and R^2^ as the model performance criteria. 

**Table 4:** Results of calibration and validation of the SWAT model for monthly flow, total suspended sediment (TSS), nitrogen (TN) and phosphorus (TP).

		Calibration				Validation			
Variable	Observed Mean	Predicted Mean	NSE	R^2^		Observed Mean	Predicted Mean	NSE	R^2^
Flow (m^3^/s)	3.31	3.00	0.52	0.55		3.83	2.18	0.60	0.76
TSS (kg/ha)	20.34	26.11	0.58	0.73		44.15	16.09	0.25	0.67
TN (kg/ha)	0.55	0.32	0.50	0.66		0.73	0.33	0.33	0.5
TP (kg/ha)	0.09	0.10	0.60	0.72		0.14	0.09	0.73	0.89

### 3.2. Performance of Management Practices

#### 3.2.1. Under Historical Climate Condition (1992–2007)

Many studies have evaluated the hydrological impacts of land use changes by hypothetically predicting the land use changes, such as conversion of the entire watershed to agricultural lands, or systematically changing land use distribution at different percentages [12,14]. Such simulation results may fail to provide an expectation on how a watershed would respond when a complex land use change occurs. This study incorporated the historical land use changes with corresponding agricultural management, and measured weather data into the model simulation to reduce the uncertainty in the evaluation of current conservation practices. Additionally, evaluating the performance of alternative conservation practices that would have further improved water quality provides more precise information on their effectiveness for making future watershed management decisions. Table 5, Table 6, and Table 7 summarize the effectiveness of 171 different management practice combinations in reducing annual pollutant losses from the watershed for total suspended sediment (TSS), total N (TN), and total P (TP), respectively. Notably, the values shown in these tables are the average annual values during the simulation period (1992–2007) and can be considered as the average watershed responses for these management practice scenarios. These simulation results for 171 scenarios were compared with the pollutant losses from the current management practice (baseline) scenario, which included various pasture management practices in the watershed from 1992 to 2007 [5]. 

The best management practice combination for mitigating TSS losses is scenario 61, which is a combination of 4.94 t/ha alum-amended litter applied in spring, no grazing and a VFS ratio of 42. The worst management practice combination is scenario 39, which is a combination of no litter application, overgrazing and no VFS. Vegetated filter strips (VFS) were the most influential management practices for reducing sediment losses. In the same field, a smaller VFS ratio indicates a larger VFS compared to a larger VFS ratio, indicating that a smaller VFS is implemented. In this study, the VFS ratios were derived using the maximum sediment loads (2,333 kg/ha/year) from the Lincoln Lake watershed under various weather conditions during a 25-year period (2004–2028). However, the maximum sediment loads (224.3 kg/ha/year for scenario 39) from the watershed over the past 16 years (1992–2007) were significantly less than the sediment loads in extreme weather conditions. Therefore, a VFS ratio of 76 could reduce sediment losses by 14.1%–23.8% compared with the baseline scenario. A VFS ratio of 42 resulted in similar sediment losses ranging from 127.1 to 141.1 kg/ha/year (Table 5) with corresponding reductions of 24.2% and 15.8%, respectively. When no VFS was implemented, overgrazing increased sediment losses from 164.7 to 224.3 kg/ha/year as the results of a loss of vegetative cover, soil compaction and reduction in infiltration. When no VFS was implemented, TSS losses generally increased with the intensity of grazing for all nutrient management scenarios. Litter application timing also affected the losses of sediment. For example, TSS losses of 197.5, 179.7, and 159.3 kg/ha/year were simulated when 4.94 t/ha alum-treated litter was applied in summer, fall and spring seasons, respectively. Spring and fall are the primary growing seasons for Bermuda grass and Fescue grass, while Bermuda was harvested twice in mid July and September. Therefore, summer application affected growth of grass to a lesser extent, and TSS losses were greater than those for spring and fall applications. Additionally, plants may experience nutrient stress when no litter is applied, resulting in less vegetated cover and increased losses of TSS.

**Table 5:** Total suspended sediment (TSS, kg/ha) losses from the Lincoln Lake watershed under 171 management practice scenarios (NG: no grazing, OG: optimum grazing, OVG: over grazing), VFS (vegetative filter strips, no VFS, VFS ratio = 42, 76) and application timing (spring, summer and fall applications), where the number indicates the litter amount in ton/acre unit with non-alum (NA) or alum (A) amended poultry litter. Bold value and underlined value indicate the best and the worst pasture management scenarios, respectively.

		VFS ratio										
		0				42				76		
		Grazing and Pasture Management										
Manure application (t/ha)-type		NG	OG	OVG		NG	OG	OVG		NG	OG	OVG
No application		187.1	195.3	224.3		133.0	134.8	141.1		134.2	136.3	144.0
Spring												
2.47A		166.1	167.8	177.0		128.4	128.7	130.4		129.1	129.4	131.3
3.71A		162.1	163.2	169.5		127.6	127.8	128.9		128.2	128.4	129.7
4.94A		159.3	160.0	164.7		**127.1**	127.2	128.0		127.6	127.8	128.6
2.47NA		168.2	170.5	182.3		128.9	129.3	131.4		129.6	130.1	132.4
3.71NA		164.4	165.9	174.7		128.1	128.3	129.9		128.7	129.0	130.7
4.94NA		161.4	162.5	169.1		127.5	127.7	128.8		128.1	128.3	129.5
Summer												
2.47A		165.3	167.9	186.4		128.5	129.0	132.6		129.2	129.7	133.8
3.71A		172.2	172.9	183.8		129.9	129.9	131.7		130.8	130.8	132.8
4.94A		197.5	198.3	210.8		135.1	135.1	137.3		137.1	137.0	139.4
2.47NA		166.4	169.2	190.1		128.7	129.3	133.4		129.4	130.0	134.8
3.71NA		164.1	166.3	181.0		128.2	128.6	131.3		128.9	129.3	132.4
4.94NA		170.0	170.5	180.0		129.4	129.4	131.0		130.2	130.2	132.0
Fall												
4.94A		179.7	180.9	187.3		131.5	131.7	132.7		132.8	133.0	134.0
6.18A		184.2	185.7	193.8		132.4	132.7	134.1		133.9	134.2	135.7
7.41A		180.0	181.4	189.6		131.5	131.7	133.2		132.7	133.0	134.7
4.94NA		167.4	168.1	172.6		129.1	129.2	129.8		129.9	130.0	130.6
6.18NA		175.3	176.1	180.1		130.7	130.8	131.4		131.8	131.9	132.5
7.41NA		181.8	182.7	187.4		132.0	132.2	132.9		133.4	133.5	134.3

The best management practice combination (*i.e.*, scenario 77) for cumulative TN losses comprises no litter application, optimum grazing and a VFS ratio of 42, while the worst management practice combination (*i.e.*, scenario 16) consists of 7.41 t/ha alum-treated litter application in the fall, no grazing and no VFS. Overgrazing decreased losses of TN from the watershed for all litter application rates, application timings, and VFS ratios (Table 6). Nutrients are normally removed from pastures by haying or by animals through grazing. When a pasture is grazed, nutrients can be returned to pasture lands via animal urine and feces excreted. Since nitrogen is usually the limiting nutrient for pasture growth, nitrogen inputs as fertilizer or manure are needed to sustain forage production. Therefore, overgrazing reduced TN losses, possibly because the amount of N removed via forage consumed by grazing animals is greater than that of N returned to the pasture in the form of animal manure. However, overgrazing increased TN losses when no litter was applied. 

**Table 6:** Total nitrogen (TN, kg/ha) losses from the Lincoln Lake watershed under 171 management practice scenarios (NG: no grazing, OG: optimum grazing, OVG: over grazing), VFS (vegetative filter strips, no VFS, VFS ratio = 42, 76) and application timing (spring, summer and fall applications), where the number indicates the litter amount in ton/acre unit with non-alum (NA) or alum (A) amended poultry litter. Bold value and underlined value indicate the best and the worst pasture management scenarios, respectively.

		VFS ratio										
		0				42				76		
		Grazing and Pasture Management										
Manure application (t/ha)-type		NG	OG	OVG		NG	OG	OVG		NG	OG	OVG
No application		3.0	3.0	3.1		2.4	**2.4**	2.5		2.4	2.4	2.5
Spring												
2.47A		3.3	3.3	3.2		2.5	2.5	2.5		2.6	2.6	2.5
3.71A		3.5	3.5	3.3		2.6	2.6	2.6		2.7	2.6	2.6
4.94A		3.7	3.6	3.5		2.7	2.7	2.6		2.7	2.7	2.7
2.47NA		3.2	3.2	3.1		2.5	2.5	2.5		2.6	2.5	2.5
3.71NA		3.4	3.4	3.2		2.6	2.6	2.5		2.6	2.6	2.5
4.94NA		3.5	3.5	3.4		2.6	2.6	2.6		2.7	2.7	2.6
Summer												
2.47A		3.6	3.6	3.5		2.7	2.7	2.6		2.7	2.7	2.7
3.71A		4.3	4.2	4.0		2.9	2.9	2.8		3.0	2.9	2.9
4.94A		5.1	5.0	4.9		3.2	3.2	3.1		3.3	3.3	3.2
2.47NA		3.5	3.5	3.4		2.6	2.6	2.6		2.7	2.7	2.6
3.71NA		3.8	3.8	3.7		2.8	2.7	2.7		2.8	2.8	2.7
4.94NA		4.2	4.2	4.0		2.9	2.9	2.8		3.0	2.9	2.9
Fall												
4.94A		5.3	5.3	5.1		3.3	3.3	3.2		3.4	3.4	3.3
6.18A		6.0	5.9	5.8		3.6	3.6	3.5		3.7	3.7	3.6
7.41A		6.5	6.5	6.3		3.8	3.8	3.7		3.9	3.9	3.8
4.94NA		4.5	4.4	4.3		3.0	3.0	2.9		3.1	3.1	3.0
6.18NA		4.9	4.9	4.7		3.2	3.2	3.1		3.3	3.3	3.2
7.41NA		5.4	5.4	5.2		3.4	3.4	3.3		3.5	3.5	3.4

The greatest TN losses were predicted for fall litter applications under all application rates. Additionally, vegetated filter strips decreased TN losses significantly and a smaller VFS ratio was more effective in reducing TN losses than greater VFS ratios.

Table 7 lists the TP losses from the watershed for 171 management practice scenarios evaluated in this study. Overall, TP losses were reduced by 43.4–68.1% compared with the baseline scenario. The worst case scenario (*i.e.*, scenario 57) is a combination of 3 tons/acre litter application, overgrazing management and no VFS. Meanwhile, the best management practice scenario (*i.e.*, scenario 58) is a combination of no litter application, no grazing and a VFS ratio of 42. 

**Table 7:** Total phosphorus (TP, kg/ha) losses from the Lincoln Lake watershed under 171 management practice scenarios (NG: no grazing, OG: optimum grazing, OVG: over grazing), VFS (vegetative filter strips, no VFS, VFS ratio = 42, 76) and application timing (spring, summer and fall applications), where the number indicates the litter amount in ton/acre unit with non-alum (NA) or alum (A) amended poultry litter. Bold value and underlined value indicate the best and the worst pasture management scenarios, respectively.

		VFS ratio										
		0				42				76		
		Grazing and Pasture Management										
Manure application (t/ha)-type		NG	OG	OVG		NG	OG	OVG		NG	OG	OVG
No application		0.6	0.6	0.8		**0.3**	0.3	0.4		0.3	0.3	0.4
Spring												
2.47A		0.7	0.8	0.8		0.3	0.4	0.4		0.4	0.4	0.4
3.71A		0.8	0.8	0.9		0.4	0.4	0.4		0.4	0.4	0.4
4.94A		0.8	0.9	0.9		0.4	0.4	0.4		0.4	0.4	0.4
2.47NA		0.8	0.8	0.9		0.4	0.4	0.4		0.4	0.4	0.4
3.71NA		0.9	0.9	1.0		0.4	0.4	0.4		0.4	0.4	0.4
4.94NA		0.9	1.0	1.1		0.4	0.4	0.4		0.4	0.4	0.5
Summer												
2.47A		0.7	0.8	0.9		0.4	0.4	0.4		0.4	0.4	0.4
3.71A		0.8	0.9	1.0		0.4	0.4	0.4		0.4	0.4	0.4
4.94A		1.0	1.0	1.2		0.4	0.4	0.5		0.4	0.5	0.5
2.47NA		0.8	0.8	1.0		0.4	0.4	0.4		0.4	0.4	0.4
3.71NA		0.9	0.9	1.0		0.4	0.4	0.4		0.4	0.4	0.4
4.94NA		1.0	1.0	1.2		0.4	0.4	0.5		0.4	0.5	0.5
Fall												
4.94A		1.0	1.0	1.1		0.4	0.4	0.4		0.4	0.4	0.5
6.18A		1.1	1.1	1.2		0.4	0.4	0.5		0.5	0.5	0.5
7.41A		1.1	1.2	1.3		0.5	0.5	0.5		0.5	0.5	0.5
4.94NA		1.0	1.0	1.1		0.4	0.4	0.5		0.4	0.4	0.5
6.18NA		1.1	1.2	1.3		0.5	0.5	0.5		0.5	0.5	0.5
7.41NA		1.3	1.3	1.4		0.5	0.5	0.5		0.5	0.5	0.6

Unlike the impact of grazing intensity on TN losses, higher grazing intensity increased TP losses from the watershed. When no VFS was present in the watershed, the TP losses ranged from 0.6 to 1.3 kg/ha/year for no grazing and optimum grazing conditions, while TP losses ranged from 0.8 to 1.4 kg/ha/year for overgrazed conditions. The impacts of VFS on TP losses were similar for VFS ratios of 42 and 76 where TP losses were reduced by 43.4–68.1% compared with the baseline scenario. Nutrient inputs from manure are usually based on meeting the N-demand of pasture; phosphorus inputs thus generally exceed the P requirement for plant growth [47,63]. Since phosphorus is easily attached to soils and transported in both soluble and sediment attached forms, high stocking rates can result in greater erosion and TP losses. Similarly, TP losses increased with increasing litter application rates. TP losses were slightly greater for summer application than for fall application due to greater precipitation and higher soil temperature, which makes phosphorus more easily attach to soils [64]. For all types of grazing management and VFS ratios, TP losses for alum-treated litter application in spring ranging from 0.3 to 0.9 kg/ha/year were less than those for the baseline (0.98 kg/ha/year). Moore [57] found that poultry litter amended with alum reduces the availability of soluble P, thus reducing the runoff losses from pasture areas. 

#### 3.2.2. Under Future Climate Conditions (2010–2069)

Table 8 summarizes the simulation results of 172 pasture management combinations under the historical climate condition, no climate change condition and six climate change conditions. Among those 171 pasture management combinations, the best pasture management combinations that result in the least pollutant losses were selected to show the maximum pollutant reduction by comparing with the water quality improvement brought by current pasture management (Table 8). The annual average TN and TP losses under different climate conditions were within a similar range of 2.4–6.5 kg/ha and 0.3–1.9 kg/ha, respectively. However, the annual average TSS losses under the no climate change condition were similar to those under historical climate conditions with a range of 127.1 and 224.3 kg/ha, while the TSS simulations under climate change conditions, ranging from 1,085.1 to 1,772.2 kg/ha, were significantly greater than the historical annual average TSS losses. 

Kay [13] compared the uncertainty sources for future climate change impacts on flood frequency in England and suggested that the uncertainty in GCMs is the major source of uncertainty in model results. The increasing magnitude of TSS losses under future climate conditions might be influenced by the uncertainty of GCM. Moreover, as extreme precipitation events increase in future climate conditions, the magnitude of peak flows were projected to increase, resulting in increases in catchment nutrient and sediment export [65,66,67,68]. Woznicki [67] also determined that a significant change in BMP performance occurred between the current climate and future climate scenarios. Our findings suggest that future climate change could significantly impact TSS losses more than nutrient losses.

**Figure 3:**
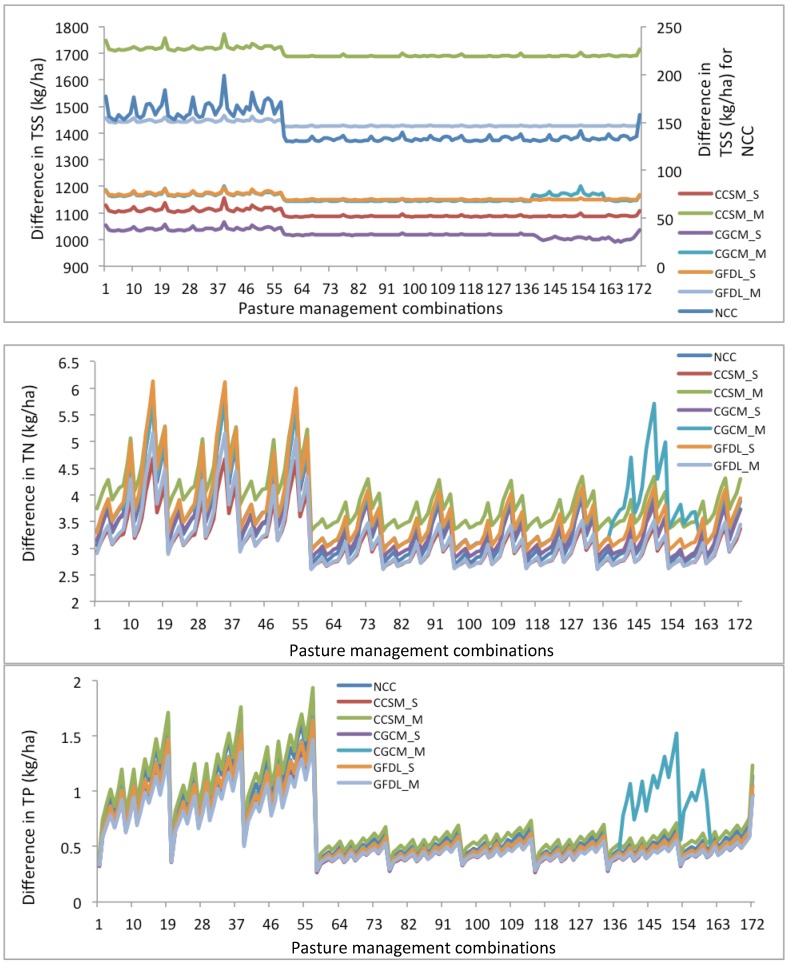
Changes in performance of 172 pasture management scenarios under historical climate and future climate conditions (Note: NCC denotes no climate change).

Those 172 pasture management combinations under no climate change and future climate conditions revealed similar patterns as the simulations under historical climate conditions (Figure 3). Under the CCSM_M condition, the TSS simulation results were the highest among other climate change conditions, ranging from 1,686.5 to 1,772.2 kg/ha. The TSS simulation results under the CGCM_S condition were the least among other climate change conditions, ranging from 990.9 to 1,066.6 kg/ha. For these three GCMs, the mid-term (2040–2069) climate conditions with more precipitation resulted in greater TSS losses than the short-term (2010–2039) climate conditions. 

**Table 8:** Range of 172 simulated pasture management performance under historical and various climate change scenarios (Note: NCC denotes no climate change condition; Difference denotes the difference between minimum and baseline simulation results).

		Historical climate	NCC	CCSM_S	CCSM_M	CGCM_S	CGCM_M	GFDL_S	GFDL_M
TSS (kg/ha)	Baseline	167.9	157.7	1,108.2	1,715.3	1,036.5	1,166.4	1,169.7	1,443.1
	Min	127.1	129.9	1,085.1	1,686.5	990.9	1,143.6	1,148.0	1,424.4
	Max	224.3	198.5	1,155.9	1,772.2	1,066.6	1,202.2	1,196.7	1,464.7
	Difference (%)	−24.3	−17.6	−2.1	−1.7	−4.4	−2.0	−1.9	−1.3
TN (kg/ha)	Baseline	3.7	3.7	3.4	4.3	3.7	3.9	3.9	3.4
	Min	2.4	2.7	2.6	3.3	2.8	3.0	2.9	2.6
	Max	6.5	6.0	4.7	5.9	5.8	5.7	6.1	5.2
	Difference (%)	−34.6	−28.6	−22.2	−22.6	−25.9	−23.8	−25.5	−24.4
TP (kg/ha)	Baseline	1.0	1.1	1.0	1.2	1.0	1.1	1.0	0.9
	Min	0.3	0.3	0.3	0.3	0.3	0.3	0.3	0.3
	Max	1.4	1.8	1.5	1.9	1.5	1.7	1.6	1.5
	Difference (%)	−68.6	−74.7	−72.5	−72.5	−72.2	−72.6	−72.5	−70.1

**Figure 4:**
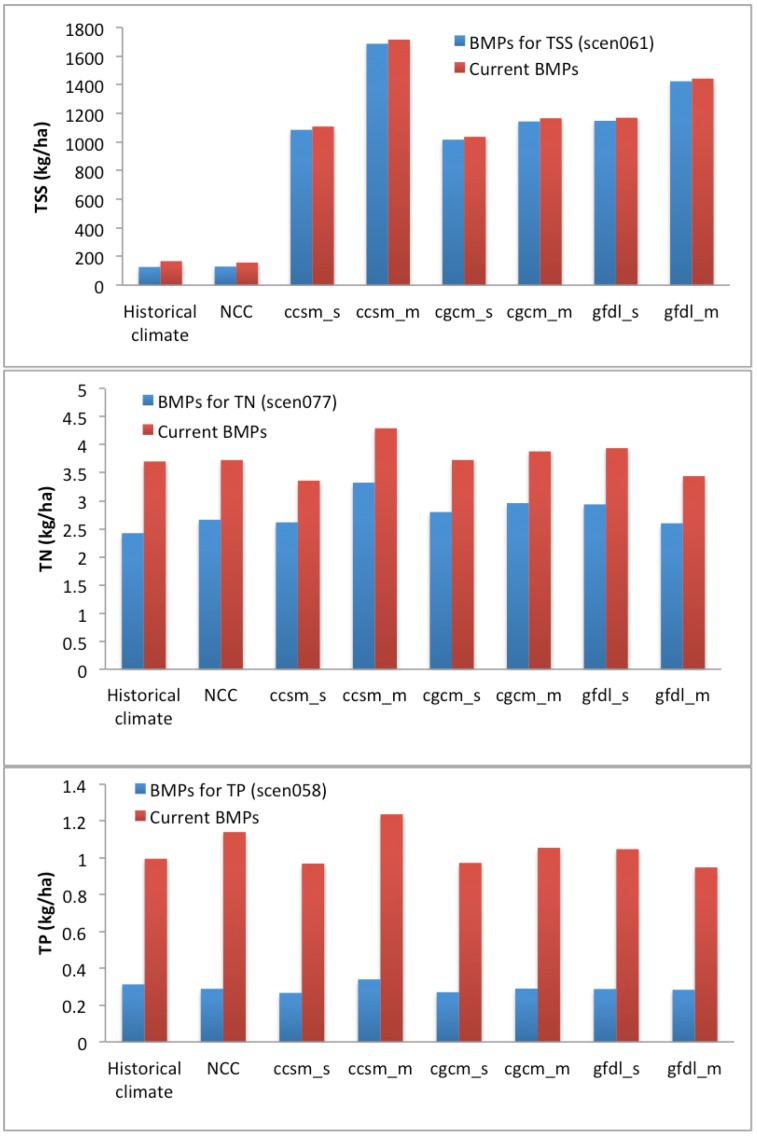
Comparison of TSS, TN and TP under different climate conditions with selected BMPs and current BMPs.

The best pasture management combination performs better than the current pasture management under historical and no climate change conditions in terms of the least TSS losses (127.1–129.9 kg/ha) and the greatest TSS reduction (17.6%–24.3%) (Figure 4). The impact of climate change on nutrient losses was expected in other studies [68,69]. Van Liew [68] concluded that TN and TP losses under the future climate scenarios are projected to be about 1.2–1.9 times and up to 1.7 times, respectively greater than the baseline for two watersheds in Nebraska. Unlike the impact of climate change on TSS losses, climate change only slightly impacted TN losses, in comparison with the simulated TN losses for the current pasture management (scen172) range of 3.4 to 4.3 kg/ha under historical and future climate change conditions. Moreover, the minimum and maximum TN losses among these 171 pasture management combinations ranged from 2.4–3.3 kg/ha and from 4.7–6.5 kg/ha, respectively, for all climate conditions. Among these climate conditions, the same best pasture management combination results in better efficiencies under the historical climate condition than under the future climate change condition. The TN reduction generally ranged from 22.2% to 25.9% for all climate change conditions. Under the CCSM and CGCM conditions, the best pasture management combination performed better in the short-term (2010–2039) than in the mid-term (2040–2069) (Figure 4). Changes in the performance of the best pasture management differ under the GFDL condition. The impact of climate change on TP losses was even smaller than that on TN losses. The TP simulations ranged from 0.9–1.2 kg/ha for the current pasture management under all climate conditions. The TP improvement from the best pasture management ranged from 68.6 to 74.7%, which is greater than TSS and TN improvements. Similar to the impact of CCSM and CGCM climate conditions on TN losses, TP losses were greater in the mid-term (2040–2069) than in the short-term (2010–2039) (Figure 4). The mid-term impact of climate change on nutrient losses was greater than the short-term impact for the Lincoln Lake watershed. Wu [70] suggested that three greenhouse gas emission scenarios (B1, A1B, and A2) for 2040 through 2069 would result in decreases in precipitation ranging from 8.5 to 9.0% and increases in air temperature ranging from 1.9 to 3.1 °C. Under these climate conditions, hydrological components in the semiarid James River Basin in the Midwestern United States could be altered considerably. Their results highlight possible risks of drought, water supply shortage, and water quality degradation in this basin. Zhang [69] also found that the simulated annual TP load shows an insignificant increasing trend with the change rate of 3.77 t per decade.

### 3.3. Hindcast of Cumulative Pollutant Losses from the Watershed

Despite the use of CEAP watersheds in several studies to evaluate the advantages of existing conservation practices [8,9,71], few of them evaluated how much conservation practices improved water quality. Especially for watersheds where only a small portion of the watershed area is managed with conservation practices, exactly how conservation practices affect water quality remains unclear [72]. Application of watershed models in evaluating certain conservation practices in past periods provides information on the effectiveness of such practices. However, assuming a constant land condition during the simulation period can increase the uncertainty of the effectiveness of conservation practices [8,73]. During the evaluation of conservation practices in the Upper Mississippi River Basin, a previous study developed a no-practice scenario to compare with the baseline scenario which includes conservation practices based on farmer survey information [1]. The difference between these two scenarios represents the cumulative benefits of conservation practices currently implemented in the watershed. Possible uncertainty in this measure in terms of the effectiveness of conservation practices is expected because the no-practice scenario was a technological step backward of conservation, and does not fully represent the previous era when conservation practices were not used. 

**Figure 5:**
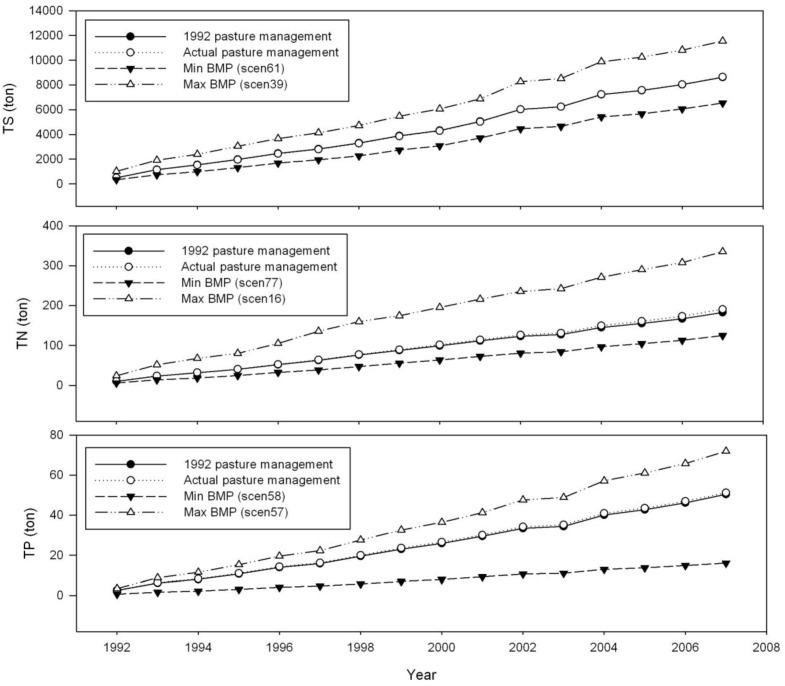
Cumulative pollutant losses for 1992 pasture management, actual pasture management (baseline), minimum and maximum pollutant losses scenarios at the Lincoln Lake watershed over the period 1992–2007.

Figure 5 shows the cumulative total suspended sediment (TSS), total N (TN) and total P (TP) losses from the watershed from 1992–2007 for four management practice scenarios. The management practices, which had the maximum and minimum cumulative constituent losses in 2007, were selected and compared with the current pasture management scenario (baseline), which is dynamic pasture management practices from 1992 to 2007, as well as the 1992 pasture management scenario, which is assumed that pasture management practices remained the same from 1992 until 2007. Details of the 1992 pasture management can be found in Chiang [27].

With insufficient nutrient supply to plant growth, less vegetated cover resulted in greater sediment losses. Higher stocking rates could result in greater sediment losses due to a decrease in infiltration of soils and an increase in soil compaction. Overgrazing should thus be avoided to reduce sediment losses from a watershed. Vegetated filter strips with a lower VFS ratio are more effective in reducing sediment losses than VFS with a greater VFS ratio. Therefore, if the best management practice combination had been implemented in 1992, a 2,104 tons cumulative TSS reduction would have been achieved by the end of 2007 compared to the baseline scenario (8,646 tons). However, sediment losses would have increased by 2,904 tons if the worst management practice combination were implemented. The cumulative TSS losses for the baseline and 1992 pasture management scenarios were similar, primarily due to that negative impacts of urbanization masked the positive impacts of conservation practices in the watershed [27].

Based on the previous comparison of 171 management practice scenarios, TN decreased with the intensity of grazing due to less nitrogen returned via feces and urine by cattle than with the nitrogen in the pasture consumed by the cattle. Additionally, TN losses increased with greater litter application rates, and TN losses incurred for alum-treated litter applications were slightly greater than untreated litter application. If the worst case scenario were implemented since 1992, the nitrogen losses would have increased by 144.3 tons by 2007 compared with the baseline scenario (190.6 tons), while the best management practice combination would have reduced TN losses by 65.9 tons. The cumulative TN losses (182.7 tons) for the 1992 pasture management scenario were slightly lower than the baseline scenario, indicating that TN losses would have been reduced if the pasture management remained the same as in 1992. This may be owing to the fact that nitrogen input from pasture management practices increased and overrode the effects of other conservation practices applied since 1994 [27].

Similar to TN losses, TP losses increased as the litter application rates increased. Since poultry litter with alum amendment has a small fraction of soluble phosphorus, alum-treated litter resulted in less mineral phosphorus losses than the untreated litter at the same application rate. Similar to the simulation results of TSS and TN losses, a smaller VFS ratio reduced more TP. TP losses would have been reduced by 35 tons by the end of 2007 compared with the baseline scenario (51.2 tons), if the best management practice combination had been implemented since 1992. However, if the worst management practice combination had been implemented since 1992, TP losses would have increased by 20.6 tons. 

### 3.4. Futurecast of Pollutant Reduction at the Sub-Basin Level

**Figure 6:**
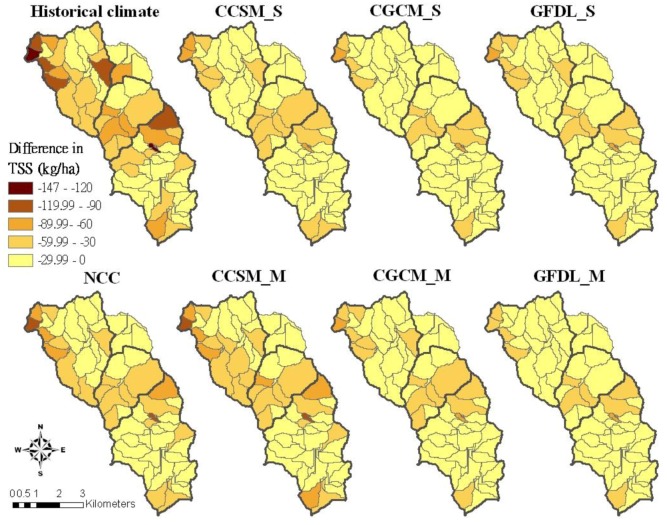
TSS reduction of the best pasture management combination (scen061) compared to the current pasture management for different climate conditions.

**Figure 7:**
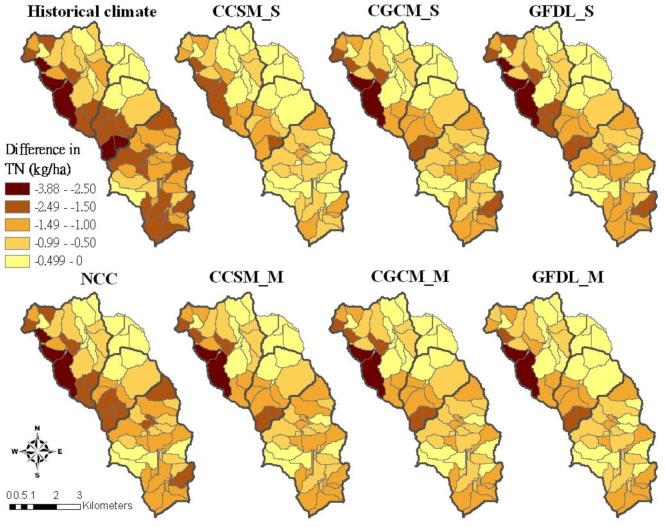
TN reduction of the best pasture management combination (scen077) compared to the current pasture management for different climate conditions.

**Figure 8:**
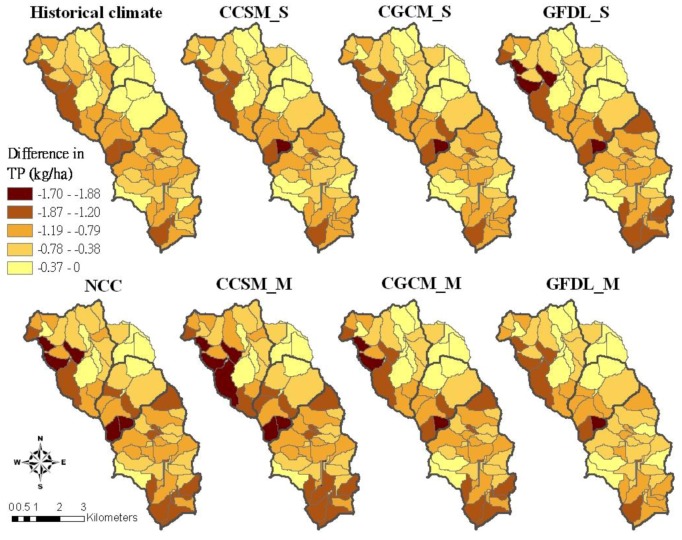
TP reduction of the best pasture management combination (scen058) compared to the current pasture management for different climate conditions.

The best management practice combination for reducing each pollutant was selected to analyze the maximum pollutant reduction at the sub-basin level that would be achieved under different future climate conditions (Table 9). Generally, significant changes in performance were more commonly observed at the field scale, while most BMPs did not affect pollution reduction at the watershed outlet [67]. The pollutant reduction was calculated as the difference between the annual average losses for the best management practice scenario and the losses for the baseline scenario. A greater difference implies a greater reduction of pollutant losses. Figure 6, Figure 7, Figure 8 show the annual average reduction of TSS, TN and TP losses for the best management practice combinations, respectively. A darker color implies a greater reduction in pollutant losses.

**Table 9:** Annual area-weighted average pollutant losses at the subbasin level with adaptation of current pasture management and the best pasture management under historical, no climate change (NCC) and climate change conditions.

	Current	scen061	Current	scen077	Current	scen058
	TSS(t/ha)	TSS(t/ha)	TN(kg/ha)	TN(kg/ha)	TP(kg/ha)	TP(kg/ha)
Historical climate	0.16	0.12	4.00	2.70	1.05	0.34
NCC	0.16	0.13	3.85	2.83	1.19	0.32
CCSM_S	1.16	1.13	3.56	2.85	1.02	0.30
CCSM_M	1.78	1.75	4.57	3.65	1.29	0.37
CGCM_S	1.08	1.06	3.87	2.97	1.02	0.30
CGCM_M	1.21	1.19	4.00	3.11	1.10	0.32
GFDL_S	1.22	1.19	4.07	3.11	1.10	0.32
GFDL_M	1.51	1.49	3.78	2.95	1.00	0.31

The annual area-weighted average of TSS losses incurred at the subbasin level would become greater in the future climate conditions, especially in the mid-term of CCSM and GFDL conditions (Table 9). This table reveals that under historical climate conditions, more subbasins would have greater annual TSS reduction of at least 90 kg/ha with the best pasture management combination than under future climate conditions (Figure 6). Although the TSS simulations of current and best pasture management practices vary under different climate conditions, the difference between those two pasture management practices could provide information about which subbasins should be given priority for BMP implementation in the watershed. Simulation results indicate that more subbasins in the Beatty Branch and Lower Moores Creek subwatersheds have greater TSS reduction, indicating that the best management practice combination was more effective in reducing TSS losses in those subbasins. The subbasins where urban area was located generally had less TSS reductions because management practices were only implemented on pasture lands. The TSS reduction was the greatest in the northwestern part of the Beatty Branch subwatershed, and the southern part of the Upper Moores Creek subwatershed. The greatest TSS reduction indicated that the maximum improvement would be found if the best management practice combination (2 tons/acre alum-treated litter in spring, no grazing and VFS ratio as 42) is implemented in the future.

With current pasture management, only under the CCSM_M and GDFL_S climate conditions, TN losses were greater than the losses under the historical climate condition (Table 9). The results are consistent with the simulation results conducted by Zhang [69] in that the simulated annual NH_4_^+^-N load into Shitoukoumen reservoir had a significant downward trend with a decrease rate of 40.6 t per decade using the SWAT model and a GCM (HadCM3). However, simulation results indicated that TN losses with the best pasture management practices would be increased in the future climate conditions compared to its performance under the historical climate condition. This finding indicates that future climate change can impact the performance of the best pasture management practices. Wu [70] concluded that the potential climate change impact would result in decreased NO3^−^N load to streams, which could be beneficial, but a concomitant increase in NO3^−^N concentration due to a decrease in streamflow likely would degrade stream water and threaten aquatic ecosystems in the watershed. A greater TN reduction was found in the western part of the Beatty Branch subwatershed (Figure 7). Under the historical climate condition, reduction in TN losses ranged from 1.5 to 3.88 kg/ha in the western part of the watershed. Moreover, a greater TN reduction would have been found in the southern part of the watershed if the best pasture management practices would have been adopted. 

In the Beatty Branch subwatershed, 2–3 subbasins would have 2.5–3.88 kg/ha of TN reduction under future climate conditions, except for the CCSM_S climate condition. The management practices impacted those subbasins under different climate change conditions in different ways, owing to dynamic nutrient management and the impacts of weather variation from 1992–2007 [5,27].

The best management practice combination improved TP reduction in the western part of the Beatty Branch subwatershed and the southern part of the Upper Moores Creek subwatershed (Figure 8). Half of the Upper Moores Creek would have at least 0.38 and 1.19 kg/ha of TP reduction in future climate conditions. In particular, we believe that, especially for the western part of the Beatty Branch subwatershed, critical subbasins have the best pasture management practices implemented; in addition, the greatest TP reduction would be found under the CCSM_M condition.

## 4. Conclusions

This study evaluated 171 pasture management combinations in the Lincoln Lake watershed for the periods 1992–2007 and 2010–2069. Due to management practices that focus on reducing different pollutants and the interactions between management practices, the best and worst management practice combinations were dissimilar for sediment, nitrogen and phosphorus. For example, overgrazing resulted in greater TSS and TP losses, but less TN losses. Intensive grazing management could increase soil compaction and decrease infiltration of soils, subsequently leading to greater TSS losses and more sediment attached P losses. Sediment losses generally decreased and nutrient losses increased with greater litter application rates. Different litter application timings influenced pollutant losses in different ways. For example, fall litter application resulted in greater TN losses while TP losses were greater for summer application. Poultry litter with alum amendment, which has a greater amount of nitrogen and less soluble phosphorus than the normal litter, resulted in greater TN losses and smaller TP losses from the watershed. Vegetative filter strips (VFS) were the most influential management practices in reducing pollutant losses, and a smaller VFS ratio (*i.e.*, the ratio of drainage area to VFS area) resulted in greater pollutant reduction. 

Compared with the baseline scenario (*i.e.*, the dynamic pasture management implemented in the watershed from 1992–2007), pollutant losses would have been reduced by 2,104, 66 and 35 tons for TSS, TN and TP by end of 2007, respectively, if the best management practice combination had been implemented since 1992. The different distribution of the cumulative reduction of pollutants during the periods of 1992–2007 and 2010–2069 revealed that different amounts of pollutants would have been or will be reduced if the best management practice combinations are implemented. Generally, BMPs under the future climate change scenarios would provide sufficient nutrient load reductions that are comparable to the respective loads simulated for the current day baseline condition. The simulation results indicated that the western part of the Beatty Branch subwatershed, as well as the northern and southern parts of the Upper Moores Creek subwatershed are the critical areas that are sensitive to climate change and must implement BMPs. 

BMPs are often implemented in a watershed without considering their watershed scale impacts or comparative analysis with other candidate BMPs. Without such an analysis, BMPs implemented in the watershed may not be able to meet the water quality goals. Results of this study demonstrate that watershed management should incorporate comparative analysis of various suites of BMPs, in addition to those implemented previously or under consideration for the future. With such an analysis, a watershed manager is more likely to achieve water quality goals or devise BMP implementation strategies in similar watersheds.

## References

[1] USDA-NRCS (Natural Resources Conservation Service) Assessment of the Effects of Conservation Practices on Cultivated Cropland in the Upper Mississippi River Basin, 2010.

[2] Gale J.A., Line D.E., Osmond D.L., Coffey S.W., Spooner J., Arnold J.A., Hoban T.J., Wimberley R.C. (1993). Evaluation of the Experimental Rural Clean Water Program; EPA-841-R-93-005.

[3] Harmel R.D., Rossi C.G., Dybala T., Arnold J., Potter K., Wolfe J., Hoffman D. (2008). Conservation effects assessment project research in the Leon River and Riesel watersheds. J. Soil Water Conserv..

[4] Webber D.F., Mickelson S.K., Ahmed S.I., Russell J.R., Powers W.J., Schultz R.C., Kovar J.L. (2010). Livestock grazing and vegetative filter strip buffer effects on runoff sediment, nitrate, and phosphorus losses. J. Soil Water Conserv..

[5] Chaubey I., Chiang L., Gitau M.W., Sayeed M. (2010). Effectiveness of BMPs in improving water quality in a pasture dominated watershed. J. Soil Water Conserv..

[6] Gitau M.W., Gburek W.J., Jarrett A.R. (2005). A tool for estimating best management practice effectiveness for phosphorus pollution control. J. Soil Water Conserv..

[7] Merriman K.R., Gitau M.W., Chaubey I. (2009). Tool for estimating best management practice effectiveness in Arkansas. Appl. Eng. Agric..

[8] Locke M.A., Knight S.S., Smith S., Cullum R.F., Zablotowicz R.M., Yuan Y., Bingner R.L. (2008). Environmental quality research in the Beasley Lake watershed, 1995 to 2007: Succession from conventional to conservation practices. J. Soil Water Conserv..

[9] Smith D.R., Livingston S.J., Zuercher B.W., Larose M., Heathman G.C., Huang C. (2008). Nutrient losses from row crop agriculture in Indiana. J. Soil Water Conserv..

[10] Yuan Y., Locke M.A., Bingner R.L. (2008). Annualized Agricultural Non-Point Source model application for Mississippi Delta Beasley Lake watershed conservation practices assessment. J. Soil Water Conserv..

[11] Bracmort K.S., Arabi M., Frankenberger J.R., Engel B.A., Arnold J.G. (2006). Modeling long-term water quality impact of structural BMPs. Trans. ASABE.

[12] Alibuyong N.R., Ella V.B., Reyes M.R., Srinivasan R., Heatwole C., Dillaha T. (2009). Predicting the effects of land use change on runoff and sediment yield in Manupali River subwatersheds using the SWAT model. Int. Agr. Eng. J..

[13] Kay A.L., Davies H.N., Bell V.A., Jones R.G. (2009). Comparison of uncertainty sources for climate change impacts: Flood frequency in England. Climatic Change.

[14] Ghaffari G., Keesstra S., Ghodousi J., Ahmadi H. (2009). SWAT-simulated hydrological impact of land-use change in the Zanjanrood Basin, Northwest Iran. Hydrol. Process..

[15] Crooks S., Davies H. (2001). Assessment of land use change in the Thames Catchment and its effect on the flood regime of the river. Phys. Chem. Earth (B).

[16] Edwards D.R., Daniel T.C., Scott H.D., Murdoch J.F., Habiger M.J., Burks H.M. (1996). Stream quality impacts of best management practices in a Northwestern Arkansas Basin. Water Resour. Bull..

[17] DeLaune P.B., Haggard B.E., Daniel T.C., Chaubey I., Cochran M.J. (2006). The Eucha/Spavinaw phosphorus index: A court mandated index for litter management. J. Soil Water Conserv..

[18] DeLaune P.B., Moore P.A., Carman D.K., Sharpley A.N., Haggard B.E., Daniel T.C. (2004). Development of a phosphorus index for pastures fertilized with poultry litter-Factors affecting phosphorus runoff. J. Environ. Qual..

[19] Vendrell P.F., Steele K.F., Nelson M.A., Cash L.W., McNew R.W. (2001). Extended Water Quality Monitoring of the Lincoln Lake Watershed; Arkansas Water Resources Center Publication No. MSC-296.

[20] Nelson M.A., Cash L.W., Trost G.K. Water quality monitoring of Moores Creek above Lincoln Lake 2006 and 2007, 2008.

[21] Arnold J.G., Srinivasan R., Muttiah R.S., Williams J.R. (1998). Large area hydrologic modeling and assessment-Part 1: Model development. J. Am. Water Resour..

[22] Gassman P.W., Osei E., Saleh A., Rodecap J., Norvell S., Williams J. (2006). Alternative practices for sediment and nutrient loss control on livestock farms in Northeast Iowa. Agr. Ecosyst. Environ..

[23] U.S. Geological Survey (USGS) (2004). USGS Geographic Data Download. http://edc2.usgs.gov/geodata/index.php.

[24] Center for Advanced Spatial Technologies (CAST) (2004). Land Use/Land Cover Data. http://www.cast.uark.edu/cast/geostor/.

[25] Gitau M.W., Chaubey I., Gbur E., Pennington J.H., Gorham B. (2010). Impacts of land-use change and best management practice implementation in a Conservation Effects Assessment Project watershed: Northwest Arkansas. J. Soil Water Conserv..

[26] 26. Pennington J.H., Steele M.A., Teague K.A., Kurz B., Gbur E., Popp J., Rodriguez G., Chaubey I., Gitau M.W., Nelson M.A. (2008). Breaking ground A cooperative approach to collecting information on conservation practices from an initially uncooperative population. J. Soil Water Conserv..

[27] Chiang L., Chaubey I., Gitau M.W., Arnold J.G. (2010). Differentiating impacts of land use changes from pasture management in a CEAP watershed using SWAT model. Trans. ASABE.

[28] Moore P.A., Edwards D.R. (2005). Long-term effects of poultry litter, alum-treated litter, and ammonium nitrate on aluminum availability soils. J. Environ. Qual..

[29] Magette W.L., Brinsfield R.B., Palmer R.E., Wood J.D. (1989). Nutrient and sediment removal by vegetated filter strips. Trans. ASABE.

[30] Lee K.H., Isenhart T.M., Schultz R.C. (2003). Sediment and nutrient removal in an established multi-species riparian buffer. J. Soil Water Conserv..

[31] Dorioz J.M., Wang D., Poulenard J. Trevisan (2006). The effect of grass buffer strips on phosphorus dynamics-A critical review and synthesis as a basis for application in agricultural landscapes in France. Agr. Ecosyst. Environ..

[32] Chaubey I., Edwards D.R., Daniel T.C., Moore P.A., Nichols D.J. (1995). Effectiveness of vegetative filter strips in controlling losses of surface-applied poultry litter constituents. Trans. ASABE.

[33] Mendez A., Dillaha T.A., Mostaghimi S. (1999). Sediment and nitrogen transport in grass filter strips. J. Am. Water Resour..

[34] Blanco-Canqui H., Gantzer C.J., Anderson S.H., Alberts E.E., Thompson A.L. (2004). Grass barrier and vegetative filter strip effectiveness in reducing runoff, sediment, nitrogen, and phosphorus loss. Soil Sci. Soc. Am. J..

[35] Dillaha T.A., Reneau R.B., Mostaghimi S., Lee D. (1989). Vegetative filter strips for agricultural nonpoint source pollution control. Trans. ASABE.

[36] Dosskey M.G., Helmers M.J., Eisenhauer D.E., Franti T.G., Hoagland K.D. (2002). Assessment of concentrated flow through riparian buffers. J. Soil Water Conserv..

[37] Dosskey M.G., Eisenhauer D.E., Helmers M.J. (2005). Establishing conservation buffers using precision information. J. Soil Water Conserv..

[38] Bren L.J. (2000). A case study in the use of threshold measures of hydrologic loading in the design of stream buffer strips. Forest Ecol. Manag..

[39] Mander U., Kuusemets V., Lohmus K., Mauring T. (1997). Efficiency and dimensioning of riparian buffer zones in agricultural catchments. Ecol. Eng..

[40] White M.J., Arnold J.G. (2009). Development of a simplistic vegetative filter strip model for sediment and nutrient retention at the field scale. Hydrol. Process..

[41] White K.L., Chaubey I. (2005). Sensitivity analysis, calibration, and validations for a multisite and multivariable SWAT model. J. Am. Water Resour..

[42] Madsen H. (2003). Parameter estimation in distributed hydrological catchment modeling using automatic calibration with multiple objectives. Adv. Water Resour..

[43] Cambell K.L., Edwards D.R. (2001). Phosphorus and Water Quality Impacts. Agricultural Nonpoint Source Pollution.

[44] Nearing M.A., Norton L.D., Zhang X. (2001). Soil Erosion and Sedimentation. Agricultural Nonpoint Source Pollution: Watershed Management and Hydrology.

[45] Nash J.E., Sutcliffe J.V. (1970). River flow forecasting through conceptual models: Part 1. A discussion of principles. J. Hydrol..

[46] Moriasi D.N., Arnold J.G., Van Liew M.W., Bingner R.L., Harmel R.D., Veith T.L. (2007). Model evaluation guidelines for systematic quantification of accuracy in watershed simulations. Trans. ASABE.

[47] Santhi C., Arnold J.G., Williams J.R., Dugas W.A., Srinivasan R., Hauck L.M. (2001). Validation of tbe swat model on a large river basin with point and nonpoint sources. J. Am. Water Resour..

[48] Van Liew M.W., Arnold J.G., Garbrecht J.D. (2003). Hydrologic simulation on agricultural watersheds: Choosing between two models. Trans. ASABE.

[49] University of Arkansas Cooperative Extension Service (UAEX) (2006). Forage and Pasture Forage Management Guides. Self-Study Guide 5: Utilization of Forages by Beef Cattle.

[50] Lowrance R., Dabney S., Schultz R. (2002). Improving water and soil quality with conservation buffers. J. Soil Water Conserv..

[51] USDA-Natural Resources Conservation Service (NRCS) (1999). CORE4 Conservation Practices Training Guide: The Common Sense Approach to Natural Resource Conservation.

[52] Naiman R.J., Decamps H., Pollock M. (1993). The role of riparian corridors in maintaining regional biodiversity. Ecol. Appl..

[53] USDA-NRCS Using RUSLE2 for the Design and Predicted Effectiveness of Vegetative Filter Strips (VFS) for Sediment. http://www.nrcs.usda.gov/Internet/FSE_DOCUMENTS/stelprdb1043476.pdf.

[54] Dillaha T.A., Hayes J.C. (1991). A Procedure for the Design of Vegetative Filter Strips.

[55] Liu X.M., Mang X.Y., Zhang M.H. (2008). Major factors influencing the efficacy of vegetated buffers on sediment trapping: A review and analysis. J. Environ. Qual..

[56] Shreve B.R., Moore P.A., Daniel T.C., Edwards D.R., Miller D.M. (1995). Reduction of phosphorus in runoff from field-applied poultry litter using chemical amendments. J. Environ. Qual..

[57] Moore P.A., Edwards D.R. (2007). Long-term effects of poultry litter, alum-treated litter, and ammonium nitrate on phosphorus availability in soils. J. Environ. Qual..

[58] Gilmour J.T., Koehler M.A., Cabrera M.L., Szajdak L., Moore P.A. (2004). Alum treatment of poultry litter: Decomposition and nitrogen dynamics. J. Environ. Qual..

[59] Lin Y.-P., Hong N.-M., Wu P.-J., Lin C.-J. (2007). Modeling and assessing land-use and hydrological processes to future land-use and climate change scenarios in watershed land-use planning. Environ. Geol..

[60] Tung C.P., Lee T.Y., Yang Y.C. (2005). Modeling climate change impacts on stream temperature of Formosan Landlocked Salmon habitat. Hydrol. Process..

[61] Tung C.P., Haith D.A. (1995). Global-warming effects on New York streamflows. J. Water Resour. Plan. Manag. ASCE.

[62] Arabi M., Frankenberger J.R., Engel B., Arnold J.G. (2008). Representation of agricultural management practices with SWAT. Hydrol. Process..

[63] Santhi C., Williams J.R., Dugas W.A., Arnold J.G., Srinivasan R., Hauck L.M. (2002). Water Quality Modeling of Bosque River Watershed to Support TMDL Analysis. Total Maximum Daily Load (TMDL) Environmental Regulations: Proceedings of the March 11-13, 2002 Conference.

[64] U.S. Environmental Protection Agency (USEPA) (2003). National Management Measures to Control Nonpoint Source Pollution from Agriculture; EPA 841-B-03-004.

[65] Mullan D.J., Favis-Mortlock D.T., Fealy R. (2012). Addressing key limitations associated with modelling soil erosion under the impacts of future climate change. Agr. Forest Meteorol..

[66] Taner M.Ü., Carleton J.N., Wellman M. (2011). Integrated model projections of climate change impacts on a North American lake. Ecol. Model..

[67] Woznicki S.A., Nejadhashemi A.P., Smith C.M. (2011). Assessing best management practice implementation strategies under climate change scenarios. Trans. ASABE.

[68] van Liew M.W., Feng S., Pathak T.B. (2012). Climate change impacts on streamflow, water quality, and best management practices for the shell and logan creek watersheds in Nebraska. Int. J. Agric. Biol. Eng..

[69] Zhang L., Lu W.X., An Y.L., Li D., Gong L. (2012). Response of non-point source pollutant loads to climate change in the Shitoukoumen reservoir catchment. Environ. Monit. Assess..

[70] Wu Y.P., Liu S.G., Gallant A.L. (2012). Predicting impacts of increased CO_2_ and climate change on the water cycle and water quality in the semiarid James River Basin of the Midwestern USA. Sci. Total Environ..

[71] McCarty G.W., McConnell L.L., Hapernan C.J., Sadeghi A., Graff C., Hively W.D., Lang M.W., Fisher T.R., Jordan T., Rice C.P., Codling E.E., Whitall D., Lynn A., Keppler J., Fogel M.L. (2008). Water quality and conservation practice effects in the Choptank River watershed. J. Soil Water Conserv..

[72] Feyereisen G.W., Lowrance R., Strickland T.C., Bosch D.D., Sheridan J.M. (2008). Long-term stream chemistry trends in the southern Georgia Little River Experimental Watershed. J. Soil Water Conserv..

[73] Kuhnle R.A., Bingner R.L., Alonso C.V., Wilson C.G., Simon A. (2008). Conservation practice effects on sediment load in the Goodwin Creek Experimental Watershed. J. Soil Water Conserv..

